# Characteristics of Older Adults Attending the Emergency Department for Suicidal Thoughts or Voluntary Intoxication: A Multicenter Retrospective Cohort Study

**DOI:** 10.7759/cureus.30428

**Published:** 2022-10-18

**Authors:** Stéphanie Boulet, Ann-Pier Gagnon, Alexandra Nadeau, Fabrice Mowbray, Éric Mercier

**Affiliations:** 1 Axe Santé Des Populations Et Pratiques Optimales En Santé, Centre de recherche du CHU de Québec - Université Laval, Quebec, CAN; 2 Axe Santé Des Populations Et Pratiques Optimales En Santé, Centre De Recherche Du Chu De Québec - Université Laval, Quebec, CAN; 3 Health Research Methods, Evidence, and Impact, McMaster University, Hamilton, CAN; 4 Axe Santé Des Populations Et Pratiques Optimales En Santé, Centre de recherche du CHU De Québec - Université Laval, Quebec, CAN

**Keywords:** mental health, intoxication, suicidal behaviours, suicidal thoughts, older adults

## Abstract

Objective

The objective of this article is to explore the characteristics of older adults visiting the ED for suicidal thoughts and/or voluntary intoxication.

Methods

All older adults (65 years or older) who visited one of the five University Hospital Center (CHU) of Quebec EDs in 2016 and who reported suicidal thoughts or intoxication in triage or received a relevant discharge diagnosis were included.

Results

A total of 478 ED visits were identified, of which 332 ED visits (n=279 patients) were included. The mean age was 72.6 (standard deviation 6.8) years old and 41.6% were female. Mood disorders (41.2%) and alcoholism (40.5%) were common. Nearly 30% of all ED visits (n=109) resulted in a referral for a mental health assessment. In the subsequent year (2017), 38.4% returned to the ED for suicidal ideations. There were 7.9% who attended the ED five times or more.

Conclusions

ED visits for suicidal thoughts and intoxication in older adults are common among men with known mood disorders or alcoholism. ED dispositions are variable, and access to a mental health assessment is not consistent.

## Introduction

Deliberate self-harm in older adults is a major public health issue, as seniors have a higher rate of suicide than any other age group [1, 2]. Furthermore, in patients ≥60 years old, the risk of suicide increases with age, with carefully planned suicides often resulting in a lethal outcome [3]. The ratio of deliberate self-harm to suicide is approximately 10:1 in older adults compared to 200:1 in teenagers [4]. For every suicide in older adults, an estimated of only two to four attempts occur [5]. Mental health disorders such as depression and anxiety are often associated with these suicides [6]. Early and accurate identification of these conditions is, therefore, the cornerstone to preventing suicide. Unfortunately, the clinicians’ ability to detect signs and symptoms of mental health disorders in older adults is often limited. In the month prior to their suicide, approximately 45% of older adults had contact with their primary care physician [7]. During these visits, mental health related complaints were rarely documented, and referrals to mental health professionals were infrequently offered. With the population aging, new strategies must be developed to enhance detection and improve the care of older adults with suicidal behaviors.

The emergency department (ED) is a crucial setting to care for vulnerable populations [8]. Overall, between 39 and 43% of all patients attended an ED in the year prior to their suicide, often for suicidal thoughts or intoxication [9, 10]. As seniors have the highest ED utilization rate [11], EDs might therefore offer a unique opportunity to intervene in older adults with suicidal behaviors. Canadian data on ED visits for suicidal behaviors in older adults are, however, scarce. Signs and symptoms of depression in seniors are under-identified by emergency clinicians [12]. Depression often presents with more subtle signs and symptoms in older adults, including physical symptoms [6]. Furthermore, when an older adult is diagnosed with depression or suicidal thoughts during an ED visit, they are less likely to have a formal mental health assessment, a treatment initiated, and/or a follow-up organized when compared to younger patients [13, 14]. The number of ED visits by older adults for mental health disorders and non-medical problems is growing, and emergency clinicians will increasingly be challenged to treat and manage mental health concerns in the ED [15]. Exploring the profiles and health service use patterns of those patients is essential to inform and improve the ED care of this vulnerable population.

The main objective of this study was to explore the characteristics of older adults who attended the ED for suicidal thoughts and/or following intoxication with the intent of self-harm. The secondary objectives were to determine the ED disposition and the proportion of patients with repeat attendances.

## Materials and methods

Ethics

This study was approved by the Centre de recherche du CHU de Quebec ethics board.

Study design, setting, and population

We conducted a retrospective cohort study using medical health records of older adults (≥65 years old) who presented to one of the five EDs of the CHU de Quebec (approximately 235 000 visits per year) between January 1st, 2016, and December 31th, 2016, for suicidal thoughts or following intoxication with self-harm intent. Inclusion was considered if suicidal thoughts and/or intoxication with self-harm intent was written by the nurse in the triage form or as the final main ED diagnosis by the emergency clinician. Suicidal thoughts and deliberate intoxication often occur concomitantly [16]. For instance, a patient can express suicidal thoughts while under the influence of alcohol or illicit drugs. It would have therefore been impractical to differentiate these two entities in the analyses. During the medical chart review, patients who presented following an intoxication without any self-harm intent (medication administration errors, chronic intoxication due to renal insufficiency, etc.) were excluded. When a patient presented to one of the five EDs more than once during the study period, each episode of care was reviewed independently.

Variables

Using a standardized data collection form, the following data were extracted from the medical chart: age, sex, Canadian Triage and Acuity Scale (CTAS), chief complaint, date and time, type of residence, medical and psychiatric comorbidities, reasons for prior ED visits, intoxication including the substance involved, and the disposition following the ED visit. We also elected to do a comprehensive review of the emergency medical records from 2014 to December 31st, 2017, to capture data concerning prior and repeat ED visitation.

Statistical analysis

We conducted a series of descriptive statistics, reporting measures of central tendency (mean, median) and frequencies using Excel (Microsoft, Redmond, Washington).

## Results

Of the 478 potentially relevant ED visits identified by our search on our administrative system, we elected to include 332 ED visits (n=279 patients) and to exclude the remaining visits due to lack of a relevant diagnosis (e.g., no intoxication or suicidal thoughts (n=52), non-intentional intoxication (n=94)). The mean age of the ED cohort was 72.6 years old (standard deviation 6.8, range 65-95), and 41.6% were female. Included patients were frequently living in a private household (45.8%), and 39.4% had at least one ED visit prior to the 2016 index ED visit for suicidal thoughts or self-harm. Approximately three-quarters (74.2%) of the patients had at least one documented mental health disorder. Mood disorders (41.2%) and alcoholism (40.5%) were frequent. In 2016, among the 279 patients included, 11.1% had more than one ED visit for suicidal thoughts or self-harm. In 2017, 38.4% had at least one new ED visit for suicidal thoughts or self-harm, including 7.9% who attended the ED ≥5 times. Characteristics of included patients and ED visits are presented in Table 1.

**Table 1:** Patient and visit characteristics ED = emergency department, CTAS - Canadian Triage and Acuity Scale

Patients characteristics (n=279)	N (%)
Age, mean (SD)	72.6 (6.8)
Age groups	
65-69	120 (43.0%)
70-74	74 (26.5%)
75-79	34 (12.2%)
80-84	33 (11.8%)
85+	18 (6.5%)
Sex (female)	138 (41.6%)
Living arrangement	
Private home or apartment	165 (59.1%)
Senior housing	61 (21.9%)
Long-term care facility	4 (1.4%)
Unknown	49 (17.6%)
Documented mental health disorders	
Alcoholism	113 (40.5%)
Substance abuse disorder other than alcohol	20 (7.2%)
Anxiety-related disorders	58 (17.5%)
Mood disorders	115 (41.2%)
Psychotic disorders	12 (4.3%)
Personality disorders	71 (25.4%)
Visit characteristics (n=332)	
Canadian Triage and Acuity Scale (CTAS)	
CTAS 1	21 (6.3%)
CTAS 2	92 (27.7%)
CTAS 3	108 (32.5%)
CTAS 4	101 (30.4%)
CTAS 5	10 (3.0%)
Time of arrival	
Day (8:01-16:00)	103 (31.0%)
Evening (16:01-24:00)	167 (50.3%)
Night (00:01-8:00)	62 (18.7%)
ED disposition	
Discharged from the ED without referral	166 (50%)
Discharged after a psychiatric consultation	61 (18.4%)
Discharged after a consultation by a medical team	12 (3.6%)
Admitted in a psychiatric unit	48 (14.6%)
Admission in a medical unit	43 (13.0%)
Died in the ED	2 (0.6%)

More than 50% of ED visits occurred during the evening, and alcohol intoxication was suspected or proven during 68.1% of ED visits. Other ingested substances included benzodiazepines (8.6%), opiates (5.8%), and acetaminophen (4.1%). There were two suicides by intoxication.

Finally, 166 patients (50.0%) were discharged by the emergency physician without a referral. One hundred and nine patients (32.8%) were referred to a psychiatrist for a mental health assessment, of which 48 were admitted to a psychiatric unit, while 55 patients (16.6%) were referred to a medical team, of which 43 were admitted on a medical unit (Table 1).

## Discussion

This retrospective cohort study found that ED visits by older adults for suicidal thoughts or self-harm by intoxication commonly involved men and patients with a prior history of mood disorders and/or alcoholism. Approximately one-third of visits resulted in a referral by the emergency clinician for a mental health assessment by a psychiatrist. About one-third of patients attended the ED more than once for a mental-health complaint.

Few studies have focused on self-harm and suicidal ideations in older adults from an ED perspective [17]. Suicide prevention often relies on a timely and effective detection of mental health disorders [18]. Even though suicidal thoughts and self-harm are complex entities involving multiple factors that are unlikely to be addressed during a single ED visit, emergency clinicians have the opportunity to identify high-risk behaviors and initiate an intervention. Following half of ED visits, patients were discharged by the emergency clinician, and mental health consultations were offered following 32.8% of all ED visits. This number was lower than anticipated. There are known barriers relative to mental health care access for older adults that could potentially have contributed to the low referral rate. For instance, depressive symptoms are often considered as normal feelings for older adults who experience multiple losses and functional decline [19]. Older adults are also frequently more reluctant to discuss mental health issues with healthcare professionals compared with younger adults. This might influence clinicians to discharge the patient without a mental-health assessment [12]. Outpatient follow-up has been shown to decrease suicidal behaviors [20].

Non-fatal self-harm is considered the strongest risk factor for subsequent suicide, and prompt intervention might mitigate the escalation to suicide [2, 21]. This might be particularly important in seniors as the lethality is higher than in younger patients [3]. In our cohort, there were two suicides by intoxication. None had a recent prior suicidal attempt. One patient was suffering from advanced cancer and was not known for any mental health disorder, while the other had no significant medical comorbidities but was treated for depression. Even though about one-third of patients had more than one ED visit for a mental-health presentation, none of these patients subsequently committed suicide, but the small sample size limits the generalizability of our findings.

ED-initiated interventions targeting older adults with suicidal thoughts and behaviors are lacking [8, 20, 22]. When compared to younger patients, older adults are more likely to report physical diseases and depression as stressors contributing to self-harm [23]. Functional impairment and physical illnesses such as cancer, neurologic disorder, and pain syndrome are strongly associated with suicidal attempts in older adults [23]. Substance use disorders and acute substance intoxication are associated with self-inflicted injuries [24]. Prior to the suicide attempt, visits were primarily for behavioral health or substance abuse problems in the ED [25]. Interventions that consider the unique characteristics of older adults, such as the burden of chronic and debilitating physical injuries, might be more useful than general interventions. The findings of this study shed light on a number of characteristics attributed to older ED patients presenting with complaints of suicidal ideation and self-harm, which can be used to inform clinical decisions.

Limitations

This study is limited by its retrospective design. Extracted data relied on the information available, and thus, some data might have been overlooked, including patients who might have been closely followed up by a mental health professional. Medical charts were reviewed for five EDs, but attendance in other EDs or healthcare facilities are possible, and our results may not be representative of provincial or national trends. Furthermore, the patients all attended an ED in the same city, making it potentially difficult to generalize the conclusions as access to mental health services (both inpatient and outpatient) might vary across institutions. Self-harm was limited to intentional intoxication. Prospective larger and multicentre studies could alleviate these limitations.

## Conclusions

We have shown that older patients presenting with suicidal ideation commonly suffer from depression and substance abuse yet are rarely referred for further treatment from the ED. Further research into the optimal ongoing treatment of this cohort of patients is indicated.
